# Prevalence and risk factors of depressive symptoms in a Canadian palliative home care population: a cross-sectional study

**DOI:** 10.1186/1472-684X-13-10

**Published:** 2014-03-17

**Authors:** Kathryn A Fisher, Hsien Seow, Kevin Brazil, Shannon Freeman, Trevor Frise Smith, Dawn M Guthrie

**Affiliations:** 1 School of Nursing, McMaster University, 1280 Main Street West, Hamilton, ON K8S 4L8, Canada; 2 Department of Oncology, McMaster University, 699 Concession Street, 4th Floor, Room 4-229, Hamilton, ON L8V 5C2, Canada; 3 Medical Biology Centre, 97 Lisburn Road, Belfast BT9 7BL, UK; 4 School of Public Health and Health Systems, University of Waterloo, 200 University Avenue West, Waterloo, ON N2L 3G1, Canada; 5 Department of Sociology, Nipissing University, 100 College Drive, Box 5002, North Bay, ON P1B 8 L7, Canada; 6 Department of Kinesiology and Physical Education, Wilfrid Laurier University, 75 University Avenue West, Waterloo, ON N2L 3C5, Canada

**Keywords:** Depression, Depressive symptoms, Palliative care patients, Depression Rating Scale, InterRAI Palliative Care assessment, Risk factors for depression, Prevalence of depressive symptoms

## Abstract

**Background:**

Depression in palliative care patients is important because of its intrinsic burden and association with elevated physical symptoms, reduced immunity and increased mortality risk. Identifying risk factors associated with depression can enable clinicians to more readily diagnose it, which is important since depression is treatable. The purpose of this cross-sectional study was to determine the prevalence of depressive symptoms and risk factors associated with them in a large sample of palliative home care patients.

**Methods:**

The data come from interRAI Palliative Care assessments completed between 2006 and 2012. The sample (n = 5144) consists of adults residing in Ontario (Canada), receiving home care services, classified as palliative, and not experiencing significant cognitive impairment. Logistic regression identified the risk factors associated with depressive symptoms. The dependent variable was the Depression Rating Scale (DRS) and the independent variables were functional indicators from the interRAI assessment and other variables identified in the literature. We examined the results of the complete case and multiple imputation analyses, and found them to be similar.

**Results:**

The prevalence of depressive symptoms was 9.8%. The risk factors associated with depressive symptoms were (pooled estimates, multiple imputation): low life satisfaction (OR = 3.01 [CI = 2.37-3.82]), severe and moderate sleep disorders (2.56 [2.05-3.19] and 1.56 [1.18-2.06]), health instability (2.12 [1.42-3.18]), caregiver distress 2.01 [1.62-2.51]), daily pain (1.73 [1.35-2.22]), cognitive impairment (1.45 [1.13-1.87]), being female (1.37 [1.11-1.68]), and gastrointestinal symptoms (1.27 [1.03-1.55]). Life satisfaction mediated the effect of prognostic awareness on depressive symptoms.

**Conclusions:**

The prevalence of depressive symptoms in our study was close to the median of 10-20% reported in the palliative care literature, suggesting they are present but by no means inevitable in palliative patients. Most of the factors associated with depressive symptoms in our study are amenable to clinical intervention and often targeted in palliative care programs. Designing interventions to address them can be challenging, however, requiring careful attention to patient preferences, the spectrum of comorbid conditions they face, and their social supports. Life satisfaction was one of the strongest factors associated with depressive symptoms in our study, and is likely to be among the most challenging to address.

## Background

Depression is one of the most common mental health problems worldwide [1]. It is also considered to be common [2], but by no means inevitable [3], in palliative care populations. Estimated prevalence rates for depression in palliative care populations vary widely, from 1 to 77% with a median of 10-20% [4-6]. Systematic reviews attribute the wide range of prevalence rates to a number of factors, including small samples, variation in assessment tools used, and classification of symptoms [6,7]. Common methodological criticisms include reliance on screening instruments that have not been validated in a palliative population, the lack of a clear definition of depression, and inclusion of somatic symptoms (e.g., weight loss) in depression scales thereby introducing symptom overlap with the effects and/or treatment of the terminal illness [5-8].

While the probable consequence of many of these weaknesses is to inflate prevalence rates [6,7], this is offset by the known barriers to detection and management of depression. The diagnosis of depression is not straightforward in older patients generally [9], who represent a significant proportion of the palliative care population. Diagnostic challenges unique to the palliative care setting also arise, such as distinguishing depression from the normal grief process or neurovegetative symptoms (e.g., sleep, appetite, concentration) that often accompany cancer and/or its treatment. Physicians may be reluctant to diagnose depression because they view treatment as adding to an already high medication burden or having limited effectiveness [4,10]. Oncologists and palliative care clinicians also cite a lack of time to perform the necessary diagnostic work as a significant barrier to diagnosing depression [4]. Yet, untreated depression reduces the quality of life of palliative patients, and has been associated with increased physical symptoms such as pain and fatigue, resulting in more hospitalizations and physician visits [11,12]. Depression is an independent risk factor for cancer mortality [13], is associated with an increased desire for death and assisted suicide [14], can reduce compliance with treatment [15,16], can decrease immune system response, and can cause patients to avoid making important health care decisions or plans for personal affairs [10]. This suggests that depression can be a very serious disorder, yet it appears to be treatable even in palliative populations [10,17-19].

Previous studies that have examined depression in palliative care patients have established associations with certain physical symptoms (e.g., sleep disorders, fatigue, dyspnea, pain, functional disability) [2,4,20,21], psychological symptoms (psychological well-being, spirituality, hopelessness, cognitive loss) [20,22,23], demographic variables (e.g., younger age, gender) [2,21,24,25], prognosis [2], prognostic awareness [26,27], social support [5,21,28,29], and caregiver distress [30,31]. However, prior research has been limited in several ways, notably the lack of sufficient sample sizes [5], samples that do not include people being treated in home settings [32], and the use of assessment tools that have not been validated in a palliative population [3,5].

To address these limitations, we conducted a study using a validated tool to screen for depression in a large sample of palliative home care patients. Specifically, we examined the prevalence and risk factors associated with depressive symptoms in a cross-sectional analysis of an existing dataset consisting of home care patients in Ontario (Canada) that were classified as palliative (or end-of-life). The aim was to assist clinicians in identifying palliative care patients at risk of depression that would most likely benefit from further assessment and intervention to address depression.

## Methods

### Study sample

The interRAI Palliative Care (interRAI PC) assessments were completed between 2006 and 2012 by trained case managers using all sources of information available at the time of assessment. Assessors compared the information to standardized coding rules and item definitions, and followed a standard protocol outlined in the interRAI PC manual [33]. The interRAI PC is a copyrighted instrument (http://www.interrai.org) designed for adults (18+) with palliative and end-of-life needs, regardless of setting (e.g., acute care, hospice, home care). The assessments were part of a pilot implementation of the interRAI PC in six of fourteen home care provider organizations in Ontario. All clients in the six sites who were classified as palliative were assessed with the interRAI PC. Patients were classified as palliative by the home care case manager if they were no longer responsive to curative treatment, considered to be dying, and the goal of care was to alleviate distressing symptoms in the last stage of their illness [34]. Informed consent was obtained from clients for participation in the pilot.

The study sample consisted of patients in the pilot who were 18+ living in Ontario, and not experiencing significant cognitive impairment (i.e., Cognitive Performance Score [CPS] < 4, see “Measures” below). Eliminating patients with significant cognitive impairment was done because neurodegeneration can interfere with patients’ emotional disposition, making it challenging for clinicians to diagnose depression [35].

### Measures

All variables used in the analyses were derived from information in the interRAI PC, which is one of a number of interRAI instruments currently used as routine assessments in countries within North America, the Nordic region, Western Europe, the Czech Republic and Japan. InterRAI instruments feature a number of consistently-defined measures and scales. This facilitates their portability across service sectors and can foster continuous improvement, especially in palliative care services, which are increasingly being delivered by a range of multidisciplinary service providers.

### InterRAI PC depression rating scale (DRS)

The dependent variable in this study was the score on the interRAI Depression Rating Scale (DRS). The DRS is an assessor-rated score created by summing the responses to the following 7 items: made negative statements, persistent anger with self or others, expressions of seemingly unrealistic fears, repetitive health complaints, repetitive non-health complaints, sad/pained/worried facial expressions, and crying and tearfulness. The four item response categories are rescaled and the responses summed to generate a DRS score ranging from 0 to 14. A score of three or higher has been shown to be predictive of a clinically-confirmed depression diagnosis [36]. The DRS has been validated against the Hamilton Depression Rating Scale, the Cornell Scale for Depression in Dementia, and psychiatrists’ ratings [36]. Unidimensionality, reliability and validity of the DRS in a palliative care population were also recently demonstrated, with the evidence including: a Mökken Scale homogeneity coefficient of 0.34, ordinal reliability measures ranging from 0.78 to 0.86, higher correlations of the DRS with mood items compared to physical function items (indicating convergent and divergent validity), and the ability of the DRS to distinguish between subgroups of patients in the expected manner [37].

### Other interRAI PC scales

The interRAI PC contains a number of embedded health index scales. For example, the Cognitive Performance Scale (CPS) can range from 0 (cognitively intact) to 6 (very severe impairment), and it has been validated against the Mini-Mental State Examination [38]. Functional performance was measured using the Activities of Daily Living Self-performance Hierarchy Scale (ADL SHS), which categorizes 4 ADLs (locomotion, eating, personal hygiene, toilet use) on a scale ranging from 0 (independence) to 6 (total-dependence in late-loss ADLs) [39]. A score of 2 or more represents a conventionally-used cut-off indicating that limited assistance is required for at least one of the ADLs. Pain was assessed using a four-point pain scale ranging from 0 (no pain) to 3 (excruciating daily pain), with a cut-off of 2 used to identify patients experiencing daily pain. The pain scale has been validated against the Visual Analogue Scale [40] and is used as an outcome quality indicator in home care settings [9]. Health instability and illness severity was measured using the Changes in Health, End-Stage disease and Signs and Symptoms (CHESS) scale. Scores range from 0 to 5, with 5 representing the highest level of health instability. The CHESS score is a very strong predictor of mortality independent of age and a number of other factors [41], and successfully predicts adverse outcomes compared to other measures [42].

### InterRAI PC items

Other independent variables were identified from a review of the palliative care literature, and included in the model subject to availability within the interRAI PC and significance in terms of their association with the DRS. An alpha level of 0.25 was used for the *χ*^2^ significance tests to identify the initial list of independent variables to guard against the omission of potentially-important variables. Additional file 1: Table S1 provides the definitions for the independent variables. Some variables were composite measures that were either summative (sleep disorders, gastrointestinal disorders) or conditional on the presence of one or more situations (caregiver distress, life satisfaction). For life satisfaction, principal component factor analysis and the Cronbach’s alpha coefficient were used to confirm the validity of combining five (conceptually broad) items into one measure. Factor analysis confirmed a 1-factor solution, all factor loadings were high (0.67-0.81), and the Cronbach’s alpha was 0.77 suggesting acceptable internal consistency.

### Statistical analysis

A multivariate logistic regression model was created using the DRS as the dichotomous dependent variable (DRS ≥ 3 versus <3) and the interRAI functional indicators and other significant items (p ≤ 0.25) as independent variables. The independent variables considered for inclusion in the regression model are listed in the first column of Table 1. All variables were categorical with the categories determined by examining logit plots and univariate statistics measuring distributional properties. The derivation and meaning of response categories for the independent variables is provided in the (Additional file 1: Table S1, second and third columns).

**Table 1:** **Item subgroups stratified by DRS**^**c **^**cutoff**

**Item**	**Categories**	**Unadjusted OR (95% CI)**^**a**^	**All patients (%) n**^**b **^**N = 5144**	**DRS < 3% (n) N = 4339**	**DRS ≥ 3% (n) N = 448**^**h**^	**p value for *χ***^**2 **^**test of Significance**	**% (n) missing**^**i**^
Age	18-64	1.8 (1.3, 2.5)	34.0% (1626)	33.0% (1432)	43.3% (194)	<0.0001	0.0% (0)
	65-74	1.3 (0.9, 1.9)	25.5% (1219)	25.5% (1107)	25.0% (112)		
	75-84	1.1 (0.7,1.5)	28.7% (1375)	29.3% (1273)	22.8% (102)		
	85+	Reference	11.8% (567)	12.2% (527)	8.9% (40)		
Gender	Female	1.2 (1.0,1.5)	50.9% (2422)	50.4% (2175)	55.6% (247)	0.0369	0.6% (30)
	Male	Reference	49.1% (2335)	49.6% (2138)	44.4% (197)		
Marital status	No partner	1.0 (0.8. 1.2)	38.7% (1785)	38.8% (1622)	38.3% (163)	0.8359	3.7% (178)
	Married or have partner	Reference	61.3% (2824)	61.2% (2561)	61.7% (263)		
Site^c^ windsor	Site 1	0.6 (0.3,1.1)	3.6% (174)	3.7% (161)	2.9% (13)	0.1079	0.0% (0)
	Site 2	0.7 (0.4, 1.0)	64.4% (3083)	64.8% (2812)	60.5% (271)		
	Site 3	0.7 (0.5, 1.2)	16.8% (802)	16.7% (726)	17.0% (76)		
	Site 4	0.9 (0.5, 1.5)	8.3% (397)	8.1% (352)	10.0% (45)		
	Site 5	1.1 (0.6, 2.2)	2.4% (116)	2.3% (100)	3.6% (116)		
	Site 6	Reference	4.5% (215)	4.3% (188)	6.0% (27)		
Co-morbidities (#)^d^ C	5+	1.8 (1.3, 2.4)	9.6% (458)	9.1% (395)	14.1% (63)	0.0003	0.0% (0)
	3-4	1.3 (1.0,1.6)	32.7% (1564)	32.4% (1405)	35.5% (159)		
	0-2	Reference	57.8% (2765)	58.5% (2539)	50.5% (226)		
Diagnosis prima	Cancer	0.8 (0.5, 1.1)	86.5% (4141)	91.6% (3791)	8.4% (350)	0.3094	0.0% (0)
	Cardiovascular	0.7 (0.4, 1.1)	4.6% (220)	92.6% (204)	7.4% (16)		
	COPD	0.7 (0.3, 1.5)	1.7% (81)	92.6% (75)	7.4% (6)		
	Other	Reference	7.2% (345)	89.1% (307)	10.9% (38)		
Prognosis	Death imminent (days) - < 6 wks	2.7 (2.0,3.8)	8.6% (358)	7.9% (295)	14.9% (74)	<0.0001	12.8% (615)
	>6 wks - < 6 mths	1.6 (1.3, 2.1)	47.6% (1987)	47.0% (1760)	53.7% (227)		
	6+ mths	Reference	42.8% (1827)	45.2% (1694)	31.4% (133)		
CHESS^e^	Moderate, Severe (2+)	3.3 (2.2, 4.8)	83.0% (3770)	81.9% (3359)	93.6% (412)	<0.0001	5.1% (247)
	None, Minimal (0,1)	Reference	17.0% (770)	18.1% (742)	6.4% (28)		
Awareness of prognosis	No	1.4 (1.1, 1.8)	45.8% (924)	44.2% (786)	52.5% (138)	0.0122	13.0%^j^ (305)
	Yes	Reference	54.7% (1116)	55.8% (991)	47.5% (125)		
Pain scale	Moderate-Severe ((2+)	2.4 (1.9, 3.0)	64.1% (3034)	62.6% (2680)	79.7% (354)	<0.0001	1.2% (59)
	None, Minimal (0,1)	Reference	35.8% (1694)	37.4% (1604)	20.3% (90)		
CPS^f^	Moderate-high impairment (2+)	2.1 (1.7, 2.6)	16.6% (796)	15.5% (671)	27.9% (125)	<0.0001	0.0% (0)
	None, Low impairment (0,1)	Reference	83.4% (3991)	84.5% (3668)	72.1% (323)		
ADL SHS^g^	Limited, Extensive (2+)	1.4 (1.1, 1.7)	37.4% (1752)	36.7% (1560)	44.0% (192)	0.0027	2.2% (103)
	Independent (0,1)	Reference	62.6% (2932)	63.3% (2688)	56.0% (244)		
Communication disorders	Yes	1.2 (1.1, 1.6)	29.0% (1374)	28.5% (1223)	33.9% (151)	0.0171	1.0% (46)
	No	Reference	71.0% (3367)	71.5% (3072)	66.1% (295)		
Sleep disorders	Severe	3.5 (2.8, 4.4)	27.0% (1245)	24.9% (1039)	48.1% (206)	<0.0001	3.7% (178)
	Moderate	2.1 (1.6, 2.8)	17.5% (808)	17.3% (723)	19.9% (85)		
	Minimal	Reference	55.5% (2556)	57.9% (2419)	32.0% (137)		
Appetite	Poor	1.7 (1.3, 2.1)	22.5% (1038)	21.5% (898)	31.8% (140)	<0.0001	3.5% (166)
	Good	Reference	77.5% (3583)	78.5% (3283)	68.2% (300)		
Dyspnea	Severe	1.4 (1.1, 1.8)	15.8% (750)	15.4% (662)	19.8% (88)	0.0301	1.1% (51)
	Moderate	1.2 (0.9, 1.4)	29.2% (1383)	29.1% (1250)	30.0% (133)		
	Minimal	Reference	55.0% (2603)	55.5% (2380)	50.2% (223)		
Gastrointestinal symptoms	Moderate-severe	1.9 (1.5, 2.3)	45.2%% (2037)	43.7% (1785)	59.0% (252)	<0.0001	5.8% (277)
	Minimal	Reference	54.8% (2473)	56.3% (2298)	41.0% (175)		
Life satisfaction	Low life-satisfaction	2.8 (2.3, 3.5)	41.0% (1846)	38.6% (1574)	64.0% (272)	<0.0001	6.0% (287)
	High life-satisfaction	Reference	59.0% (2654)	61.4% (2501)	36.0% (153)		
Living alone	Living alone	1.3 (1.0, 1.7Q)	19.3% (892)	19.6% (822)	16.0% (70)	0.0670	3.3% (157)
	Living with others	Reference	80.7% (3738)	80.4% (3370)	84.0% (368)		
Supportive family	No strong family support	1.6 (1.1, 2.4)	4.7% (222)	4.5% (191)	7.0% (31)	0.0170	1.1% (53)
	Strong, supportive family	Reference	95.3% (4512)	95.6% (4098)	93.0% (414)		
Caregiver distress	Caregiver exhibits signs of distress	2.7 (2.2, 3.3)	23.3% (1063)	21.3% (878)	42.2% (185)	<0.0001	4.7% (227)
	Caregiver does not exhibit signs of distress	Reference	76.7% (3497)	78.7% (3244)	57.8% (253)		

^**a**^95% CI straddling 1.0 are not statistically significant.

^b^Subgroup totals are the total sample (n = 5144) less clients where DRS or item was missing.

^c^All 6 sites are located in Ontario, Canada; site names not shown to protect patient confidentiality.

^d^Includes primary diagnosis.

^e^CHESS = Change in Health, End-Stage and Disease Symptoms and Signs.

^f^CPS = Cognitive Performance Scale.

^g^ADL SHS = Activities of Daily Living Self-performance Hierarchy Scale.

^h^Corresponds to a prevalence of depressive symptoms of 9.4% [448/(4339 + 448)×100].

^i^Expressed as the number missing for this item divided by the number of clients with a non-missing DRS score (4787 of the 5144 clients in the total sample), multiplied by 100.

^j^Expressed as the number of clients missing for this item divided by the number of clients with non-missing DRS scores and a prognosis < 6 months (n = 2345), multiplied by 100.

Multicollinearity was assessed using polychoric correlations because these are preferred as a measure of association for ordinal data [43,44]. A cutoff of 0.40 was used to identify variable pairs where elimination of one item was required to avoid mulit-collinearity problems. The following variable pairs exceeded the cutoff:

• prognosis with the CHESS score;

• prognosis and the CHESS score with the ADL SHS score and appetite problems;

• the CHESS score with dyspnea;

• the CPS score with communication problems and the ADL SHS score; and

• marital status with living alone.

Prognosis and the CHESS score are both mortality measures. CHESS was retained in the model instead of prognosis because it has been validated and displays less assessor variability in use and judgement [41]. Dyspnea, appetite problems and the ADL SHS score were eliminated because each of these directly relates to items used in calculating the CHESS score [41]. The CPS score was retained in the model instead of communication problems because the validity and reliability of the former is well established [38], and many studies report an association between cognitive impairment and depression [9,45,46]. Living alone was retained in the model instead of marital status because of the strong bi-variate association it shows with the DRS (Table 1), and because it better captures the extent of social isolation, which has been long recognized as a risk factor for depression [47,48].

The impact of missing data was also considered. Although the extent of missing data for most items is below 5% (Table 1), restricting the analysis to clients having a response for all variables included in the model (a complete case analysis) reduces the sample from 5,144 to 3,734, a reduction of 27%. While a complete case approach is often used in health research [49], other methods are receiving increased attention with the choice of method depending on the pattern of missing data and the mechanisms causing it [50]. We believe that our missing data reflect a random process rather than systematic bias. However, we cannot be certain which of the three randomness patterns described in the literature applies to our data: missing completely at random (MCAR), missing at random (MAR), or missing not at random (MNAR) [50]. There is no universal method of handling MNAR, but the pattern is rare [51]. MCAR has been found to be insensitive to the method of handling missing data [50], but this pattern is also rare [51,52]. MAR characterizes most missing data [51,52], and multiple imputation is recommended for this pattern because it leads to unbiased results with correct standard errors [50,52]. We performed multiple imputation, and provide these results alongside those for the complete case analysis, as has been previously recommended [53]. Logistic regressions were performed using backward selection and a significance level of 0.05 for retaining model variables. Risk factors were considered significant if they were selected in at least 50% of the logistic regressions (i.e., 50 regressions were run, one for each of the 50 imputations conducted, with factors considered significant if they were retained in at least 25 runs). Multiple imputation used continuous-based imputation with rounding [54] and the results for 50 imputations were pooled using normalizing transformations [55]. Imputation included the outcome variable (DRS) [56] and all independent variables left after addressing the multi-collinearity concerns discussed above.

A special sub-analysis was undertaken to explore whether prognostic awareness was a potential risk factor for depression. This analysis employed a smaller sample consisting of patients with an estimated prognosis of less than six months, because prognostic awareness in the interRAI PC pertains only to this subgroup. We also tested whether the effect of prognostic awareness on the DRS was mediated by life satisfaction, using the methodology recommended by Frazier et al. [57]. Mediation was examined because of the complex way that prognostic awareness appears to interact with acceptance in shaping depression [27]. In particular, we were interested in whether life satisfaction (which includes acceptance and optimism) mediated the relationship between prognostic awareness and the DRS, in light of the research linking “peaceful awareness” with more positive end-of-life outcomes [3,27].

It is recommended that the goodness-of-fit of prognostic models be assessed using measures of both discrimination and calibration [58,59]. The c statistic, which corresponds to the area under the receiver operating characteristic (ROC) curve, was chosen as the measure of discrimination, and the Hosmer and Lemeshow statistic was chosen to assess calibration. A c statistic of 0.70 or greater indicates good sensitivity and specificity, and a Hosmer and Lemeshow statistic that is small with a large p value indicates acceptable model fit.

SAS Version 9.2 was used for all of the statistical analyses (http://www.sas.com). The study was approved by the Office of Research Ethics at the University of Waterloo. This institution is responsible for managing and controlling access to the data used in this study.

## Results

### Sample characteristics

Sample characteristics are provided in Table 1. Two-thirds of the sample was over the age of 65, with an average age of 70.0 years and a range of 19.6-107.2 years. The study sample was evenly distributed by gender (49.1% male), and over half (61%) were currently married. The prevalence of symptoms of depression was 9.4% in the full sample (patients with DRS ≥3, footnote h of Table 1). Eighty-three percent of the sample had a CHESS score of 2 or more, with this high percentage reflecting the severity of illness characterizing this palliative population. Sixteen percent of the sample had a CPS score of 2 or more, which is approximately equal to a score of 19.2 on the Mini-mental State Examination [38]. The assessor-reported item on prognostic awareness, which applies to patients with an estimated prognosis of less than six months, indicated that just over half of these patients were aware of their prognosis. Thirty-seven percent of the sample experienced at least a moderate level of functional impairment, almost two-thirds had daily pain, and 42% had at least 3 co-morbidities, with cancer being the main diagnosis (86.5%). One quarter of the patients reported that their caregivers were distressed, although the vast majority (95%) reported having a supportive family.

### Independent variables for logistic regression

Table 1 provides the bivariate associations between the independent variables and the DRS (ORs and 95% CI, *χ*^2^ p values). The DRS was significantly (p ≤ 0.05) associated with the majority of items. The proportion of patients with DRS scores ≥ 3 was higher for patients who: were younger, female, had with more co-morbidity, pain, cognitive impairment, health instability (CHESS), needed more assistance with ADLs, sleep disorders, appetite problems, gastrointestinal problems, communication problems, dyspnea, and caregiver distress. On the other hand, the proportion of patients with DRS scores ≥3 was lower for patients with an estimated prognosis of more than 6 months, high life satisfaction and more family support. Among those with an estimated prognosis of less than six months, prognostic awareness was associated with lower DRS scores. There is little evidence of the DRS varying by marital status, site, or living arrangement (alone versus with others). There were also no significant differences in the DRS across diagnostic categories, consistent with a recent study by Steinhauser et al. [60].

### Risk factors associated with depressive symptoms

Table 2 provides the logistic regression results for the complete case and multiple imputation models. The results from the two methods were similar for the risk factors selected into the models and the relative strength of their association with the DRS. Both methods found that life satisfaction had the strongest association with the DRS, and both selected the following six risk factors: life satisfaction, CHESS, sleep disorders, pain, caregiver distress and gender. The multiple imputation analysis identified two additional risk factors associated with the DRS - cognitive impairment and gastrointestinal symptoms. The c statistic was above 0.70 for the complete case and all multiple imputation models, indicating good sensitivity and specificity. The Hosmer and Lemeshow statistic for the complete case model was relatively small with a large p value (6.97; p = 0.54), indicating model acceptability. For the multiple imputation models, this statistic showed much more variation compared to the c statistic, as expected [59], but in all cases the statistic indicated acceptable model fit.

**Table 2:** **Risk factors associated with depressive symptoms (DRS 3+) comparison of complete case and multiple imputation analyses (Model with CHESS**^**b **^**as Mortality Measure)**

**Independent variable**	**Complete case analysis (n = 3734) Adjusted odds ratio (95% confidence limit)**	**Multiple imputation (n = 5144) Pooled odds ratios (Pooled 95% confidence limit)**
**Life satisfaction**^**a**^		
Low	3.070 (2.37-3.98)	3.01 (2.37-3.82)
High	Reference	Reference
**CHESS**^**b**^		
2+	2.88 (1.79-4.62)	2.122 (1.42-3.18)
0-1	Reference	Reference
**Sleep disorders**^**c**^		
Severe	2.78 (2.14-3.60)	2.56 (2.05-3.19)
Moderate	1.52 (1.10-2.11)	1.56 (1.18-2.06)
Minimal	Reference	Reference
**Pain scale**		
2+	2.18 (1.64-2.89)	1.73 (1.35-2.22)
0-1	Reference	Reference
**Signs of caregiver distress**		
Yes	1.93 (1.51-2.45)	2.01 (1.62-2.51)
No	Reference	Reference
**Gender**		
Female	1.42 (1.13-1.79)	1.37 (1.11-1.68)
Male	Reference	Reference
**Cognitive impairment**		
CPS^d^ 2+	N/S^e^	1.45 (1.13-1.87)
0-1		Reference
**Gastrointestinal symptoms**		
Moderate-severe	N/S^e^	1.27 (1.03-1.55)
Minimal		Reference
**Goodness of fit**		
C statistic	0.77	0.77 (0.75-0.77)^f^
Hosmer & Lemeshow -*χ*^2^	6.97	5.27(1.5-14.1)^f^
-p	0.54	0.73 (0.08-0.99)^f^
-df	8	8 (8-8)^f^

^a^“High” life satisfaction indicates that the patient has all 5 of the following conditions present: a sense of completion, a sense of progress towards completion, acceptance of the situation, inner strengths that can be encouraged/nurtured AND a positive outlook. “Low” life satisfaction” indicates that one or more of the 5 conditions are absent in the patient.

^b^CHESS = Changes in Health and End-Stage Disease Signs and Symptoms.

^c“^Sleep disorders” is a composite (summative) measure based on two items: too little sleep (5 severity levels ranging from 0 to 4) and too much sleep (5 severity levels ranging from 0 to 4). “Severe” sleep disorders represent scores of 4+, “moderate” are scores of 2 or 3, and “minimal” are scores of 0 or 1.

^d^CPS = Cognitive Performance Scale.

^e^N/S = Not significant at alpha = 0.05 level.

^f^Mean (Minimum-Maximum).

**Figure 1:**
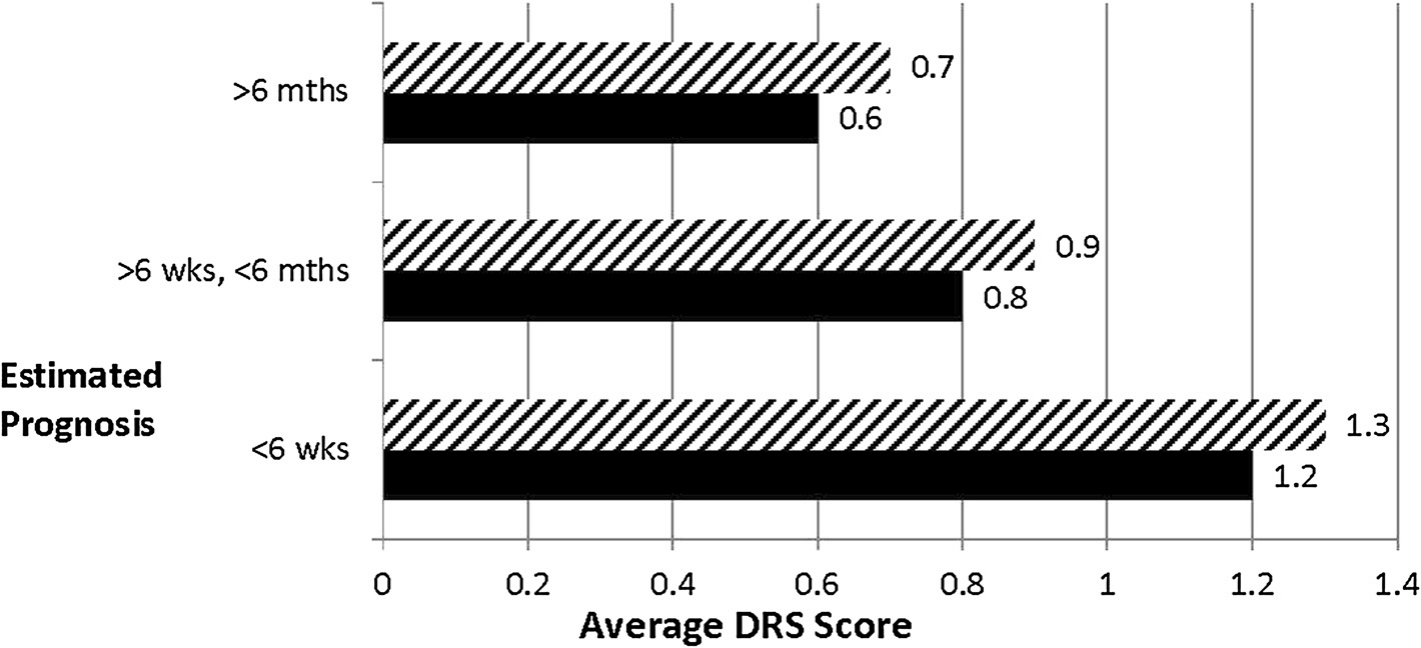
**Average Depression Rating Scale (DRS) scores by estimated prognosis.** (Males versus Females), Legend: Female , Male .

**Figure 2:**
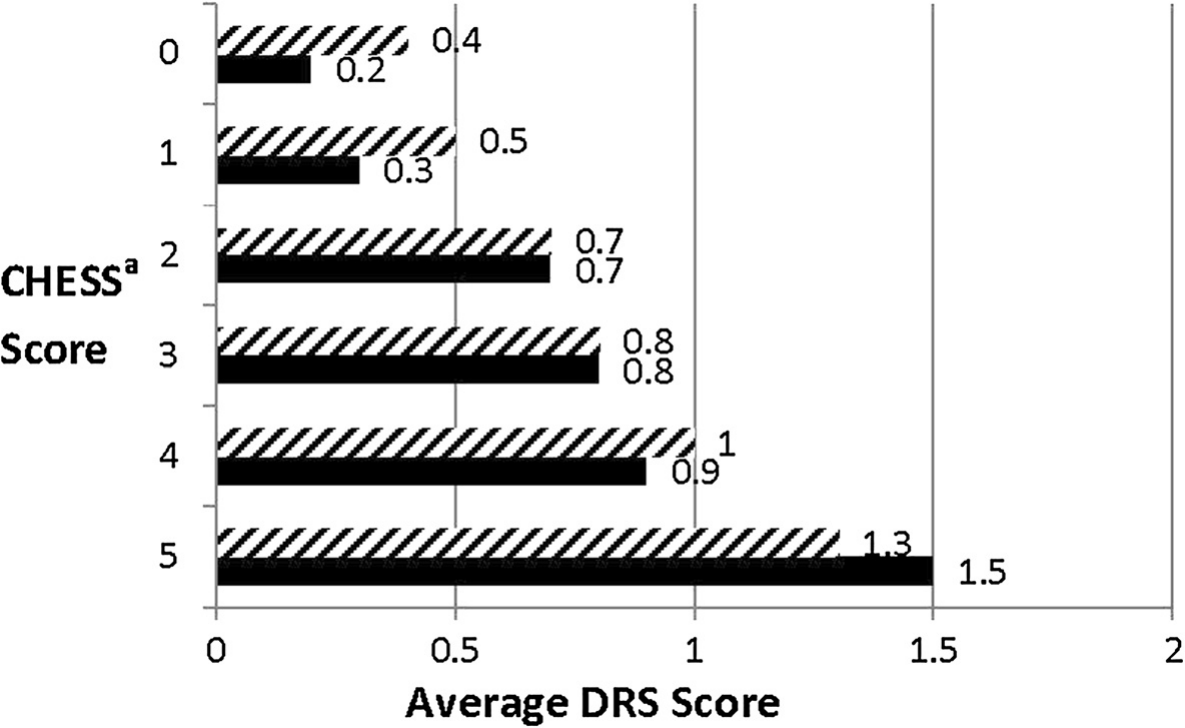
**Average Depression Rating Scale (DRS) scores by CHESS**^**a **^**Scale Score.** (Males versus Females), Legend: Female , Male . ^a^Changes in Health and End-Stage Disease Signs and Symptoms.

We note that prognosis is closely related to CHESS and performs a similar function in the model – i.e., if prognosis is included instead of CHESS, it is a significant risk factor along with the seven other variables selected into the CHESS model (see Table 2). The prognosis model also identifies one additional risk factor, number of co-morbidities, although the OR is significant for the highest category only (5+). These results reflect the conceptual overlap between CHESS and prognosis and the strong relationship both show with the DRS (see Figures 1 and 2).

We also examined whether prognostic awareness was a risk factor associated with depressive symptoms. We used multiple imputation, and ran the logistic regression for patients with an estimated prognosis of less than six months (n = 2547). Table 3 (last column) provides the regression results for the model including CHESS and prognostic awareness, and shows that the results were similar to the model including CHESS only (first column). An important finding was that prognostic awareness was *not* a risk factor associated with depressive symptoms. We also found that life satisfaction met Frazier et al.’s [57] four mediation conditions, specifically: significant relationships existed between prognostic awareness and both the DRS (pooled p = 0.03) and life satisfaction (pooled p < 0.0001), a significant relationship existed between life satisfaction and the DRS (pooled p < 0.0001), and the relationship between prognostic awareness and the DRS was significantly reduced once life satisfaction entered the model (pooled z statistic for mediation effect = 6.82, which exceeds 1.96, the p = 0.05 significance level). Therefore, prognostic awareness may not be a significant risk factor because life satisfaction mediates its effect on depressive symptoms. However, prognostic awareness may have an important *indirect* effect on depressive symptoms, given the nature and significance of its relationship with life satisfaction. For example, our data indicate that life satisfaction was significantly stronger among those that were aware of their prognosis (*χ*^2^ = 98.11, p < 0.0001).

**Table 3:** Risk factors associated with depressive symptoms (DRS 3+) comparison of multiple imputation analyses models

**Independent variable**	**Model with CHESS**^**b **^**(n = 5144) Pooled odds ratio (Pooled 95% confidence limit)**	**Model with Prognosis (n = 5144) Pooled odds ratios (Pooled 95% confidence limit)**	**Model with CHESS**^**b **^**and prognostic awareness (n = 2547) Pooled odds ratios (Pooled 95% confidence limit)**
**Life satisfaction**^**a**^			
Low	3.01 (2.37-3.82)	2.58 (1.95-3.42)	3.62 (2.70-4.85)
High	Reference	Reference	Reference
**CHESS**^**b **^**or Prognosis**			
- Imminent (days)	-	1.97 (1.32-2.94)	-
2+ - < 6 weeks	2.122 (1.42-3.18)	1.47 (1.13-1.91)	1.71 (1.05-2.80)^g^
0-1 -6 + weeks, < 6mths	Reference	Reference	Reference
**Sleep disorders**^**c**^			
Severe	2.56 (2.05-3.19)	2.67 (2.03-3.52)	2.56 (1.93-3.39)
Moderate	1.56 (2.06-1.18)	1.82 (1.31-2.53)	1.35 (0.94-1.96)
Minimal	Reference	Reference	Reference
**Pain scale**			
2+	1.73 (1.35-2.22)	1.69 (1.25-2.28)	2.09 (1.52-2.87)
0-1	Reference	Reference	Reference
**Signs of caregiver distress**			
Yes	2.01 (1.62-2.51)	1.71 (1.29-2.26)	1.41 (1.08-1.85)
No	Reference	Reference	Reference
**Gender**			
Female	1.37 (1.11-1.68)	1.47 (1.14-1.90)	1.41 (1.09-1.81)
Male	Reference	Reference	Reference
**Cognitive impairment**			
CPS^d^ 2+	1.45 (1.13-1.87)	1.71 (1.12-2.62)	1.39 (1.04-1.85)
0-1	Reference	Reference	Reference
**Gastrointestinal symptoms**			
Moderate-Severe	1.27 (1.03-1.55)	1.37 (1.06-1.76)	N/S^e^
Minimal	Reference	Reference	
**# Co-morbidities**			
5+	N/S^e^	1.79 (1.24-2.59)	N/S^e^
3-4		1.17 (0.90-1.51)	
0-2		Reference	
**Supportive family**			
No strong family support	N/S^e^	N/S^e^	1.99 (1.20-3.30)
Strong family support			Reference
**Goodness of fit**			
C statistic (ROC curve)	0.77 (0.75-0.77)^f^	0.75 (0.74-0.76)^f^	0.76 (0.75-0.76)^f^
Hosmer & Lemeshow -*χ*^2^	5.27(1.5-14.1)^f^	7.89 (2.2-16.9)^f^	9.25 (3.0-16.9)^f^
-p	0.73 (0.08-0.99)^f^	0.49 (0.03-0.98)^f^	0.37 (0.03-0.93)^f^
-df	8 (8-8)^f^	8 (8-8)^f^	8 (8-8)^f^

^a^“High” life satisfaction indicates that the patient has all 5 of the following conditions present: a sense of completion, a sense of progress towards completion, acceptance of the situation, inner strengths that can be encouraged/nurtured AND a positive outlook. “Low” life satisfaction” indicates that one or more of the 5 conditions are absent in the patient.

^b^CHESS = Changes in Health and End-Stage Disease Signs and Symptoms.

^c“^Sleep disorders” is a composite (summative) measure based on two items: too little sleep (5 severity levels ranging from 0 to 4) and too much sleep (5 severity levels ranging from 0 to 4). “Severe” sleep disorders represent scores of 4+, “moderate” are scores of 2 or 3, and “minimal” are scores of 0 or 1.

^d^CPS = Cognitive Performance Scale.

^e^N/S = Not significant at alpha = 0.05 level.

^f^Mean (Minimum-Maximum).

^g^Assumes a CHESS cutoff of 3+ versus <3 (to improve discriminatory power in this smaller sample focused on patients with prognosis < 6 months).

## Discussion

The prevalence of depressive symptoms was 9.8% in the complete case and multiple imputation samples (9.4% in full sample). This may underestimate the true rate, given that depression is often under-diagnosed in palliative populations [1], and the exclusion of somatic symptoms from the DRS may miss patients with genuine physical symptoms of depression. However, our results show that depressive symptoms are not inevitable or integral to the dying process [3,22]. While prevalence rates in palliative patients are typically higher than the 2-5% found in the general population [61], a recent review of depression in cancer patients [8] cited lower rates similar to those reported using the DRS in home care [62]. Also, few studies have examined depression in palliative patients treated at home, as we have done. One study reported a depression rate of 13% in home care patients compared to 33% for those treated in the hospital [32], but more research is needed to better understand mental health outcomes in home care settings.

Regarding the risk factors associated with depressive symptoms, complete case and multiple imputation analyses agreed on the selection of six factors: life satisfaction, mortality (measured by CHESS or prognosis), sleep disorders, pain, caregiver distress and gender. The factors other than gender and mortality are amenable to clinical intervention, thus we focus on these. Life satisfaction was the risk factor with the strongest association and, in this study, is a multidimensional construct that includes prognostic acceptance, sense of completion of personal goals/responsibilities, possession of inner strengths, and possession of a positive outlook . It is not surprising that the last two constructs, positive outlook and inner strength, are associated with fewer depressive symptoms. There is also evidence that the other constructs are linked to depression. A study of 381 palliative cancer patients found that those who did not accept their prognosis were more likely to have a diagnosis of depression or anxiety disorder [29,63]. Breitbart [64] sees acceptance of death as critical to many outcomes in palliative patients, and as a result, he recommends that this be a key goal of palliative care programs. Achieving a sense of completion of life goals/responsibilities was identified as among the most important attributes of preparing for end-of-life by patients, families, caregivers and health care providers [65]. Some psychotherapeutic interventions have shown promise in helping patients achieve a sense of completion, including the Outlook intervention [66] and dignity therapy [67]. Studies suggest that treatment control may be a key factor in maintaining continued optimism [68], suggesting that clinicians should emphasize the positive aspects of treatments.

Physical symptoms such as sleep disorders, pain and gastrointestinal disorders (identified in the multiple imputation analyses) were also risk factors associated with depressive symptoms in our study. Other research confirms the linkage of these symptoms with depression [2,5,12,20,21,69,70]. Further evidence of the association between sleep disturbance and depression comes from the clustering the former shows with depression [71], and the widespread recognition of it as a symptom of depression, leading to its inclusion in depression scales like the Beck Depression Inventory II (tiredness/fatigue) [72] and the Hamilton Depression Scale (insomnia) [73]. The cross-sectional nature of our study does not allow for the determination of directionality, therefore, physical symptoms may be the reason patients become depressed, or depressed patients may focus more on their physical symptoms. Other studies confirm this directional uncertainty, particularly for sleep and gastrointestinal disorders [4,5,74]. However, physical symptoms are also intrinsic burdens normally treated to alleviate suffering in palliative patients, regardless of their potential impact on depression. The challenge is choosing medications to treat physical symptoms with careful regard to the patient’s comorbid conditions and the goals of the palliative care program [75]. For example, a review of existing medications is one of the first things done in treating cognitive impairment, a symptom that is like sleep, pain and gastrointestinal disorders in that it is often present in palliative patients [71] and among the risk factors for depressive symptoms [75,76].

Caregiver distress was a risk factor in our study. Other studies using the DRS as a measure of depression have found the same result [77,78]. The broader research on depression and dementia indicates that patient depression is one of the main causes of caregiver stress [79]. Some studies have shown that behavioral interventions that target the patient and include caregiver participation can reduce depression in both groups [35]. Other studies show that the provision of support to caregivers to improve their coping strategies, with or without interventions for the patient, positively influenced the quality of life of dementia patients [80]. Higher odds of distress have also been observed in caregivers that provide more informal support [81], which is the case in our sample too, and suggests that providing instrumental support to caregivers and integrating informal and formal services may reduce caregiver distress.

Cognitive impairment was associated with depressive symptoms in the multiple imputation but not complete case analyses. Multiple imputation, by correcting for increased chance variation in complete cases, should generate more unbiased, and thus reliable, results [54]. There is also considerable evidence from other studies that cognitive impairment and depression are syndromes that co-exist, particularly in older adults and/or terminally-ill [9,35,45,71,75,82]. The direction of the association between the two is what remains unclear, because both have overlapping symptoms, reciprocal effects and shared etiologies [35,79,83]. This nevertheless suggests that treating cognitive impairment may reduce depression. Non-pharmacologic methods aimed at alleviating precipitating factors are recommended as the first form of intervention to treat cognitive impairment, because they have little to no downside and show high rates of reversibility in some groups (e.g., 50% reversibility in patients with advanced cancer) [74-76]. Precipitating factors include dehydration, poor nutrition, inadequate pain control, positioning that causes pressure ulcers or thrombosis, poor lighting, sleep disruption, high noise levels, absence of orienting influences, and lack of family involvement in patient care [76]. Pharmacologic interventions are also routinely used, even though no antipsychotic drugs have yet been approved by the US Food and Drug Administration for treatment of cognitive impairment [74].

Finally, we note that prognostic awareness was not a risk factor for depressive symptoms in the multivariate model, despite its strong bi-variate association with the DRS, and its linkages with depression in other studies [26]. Life satisfaction was found to mediate the main effect of prognostic awareness on the DRS. However, there may be an indirect impact of prognostic awareness on the DRS through its relationship with life satisfaction. This relationship in part reflects the dependency of prognostic acceptance (a component of life satisfaction) on awareness, since people need to be aware of something in order to accept it. However, the relationship may be more complex and involve other factors (e.g., spirituality, existential distress). More research is needed, perhaps using path analysis, to better understand the complex relationship between depression, prognostic awareness, and acceptance and what influence each of these has on mental health.

We acknowledge several limitations that influence the interpretation of the study results. The cross-sectional design is one of this study’s primary limitations as it does not allow us to determine causality. Future studies should use longitudinal data where possible. The independent variables included in the analysis were also restricted to those available from the interRAI PC. While most of the potential risk factors identified in the literature were captured in the assessment, some were unavailable, including: financial concerns, spirituality and existential distress (available but low response), and history of patient/family depression [21]. This limitation also affected our ability to fully explore the significance of prognostic awareness, because this interRAI PC item pertained to a smaller sample of patients. The resulting smaller sample may have failed to capture the full variation in some variables and may be subject to ascertainment bias (e.g., the factors shaping prognostic awareness and its association with the DRS may be different in patients having a shorter prognosis). We also did not employ diagnostic interviews (the “gold standard”) to identify depression. However, the DRS has been shown to be reliable for use as a clinical screen for depression, having been validated against psychiatric diagnoses [36] and validated in the palliative care population. We did not have information on medication use, thus the prevalence rate of depressive symptoms observed in our study may underestimate the true underlying rate due to patients’ use of antidepressants.

Despite these limitations, this study has a number of strengths. One strength is the large sample size, which enhances the reliability of our results and overcomes the high non-participation rates in other studies [6]. We also included a large number of covariates, which helps to identify the most significant risk factors for depressive symptoms, and can assist clinicians and care providers in understanding how best to screen for and treat depression in this population.

## Conclusions

This paper examined the covariates associated with depressive symptoms in palliative home care patients. While more research is needed to confirm our findings and determine causality, the results nevertheless highlight potential risk factors, most of which are amenable to clinical intervention and emphasized in palliative care programs. For example, pain, sleep and gastrointestinal disorders were significant risk factors in our study, and most palliative care programs aim to alleviate these and physical suffering generally. The challenge is treating physical symptoms concomitantly with the comorbidities often seen in palliative patients, such as cognitive impairment (also a risk factor in our study) that is caused or worsened by medications used to treat physical symptoms. Another key implication of our study’s results is providing coping-strategy-instrumental-based support to caregivers. Though often overlooked in traditional medical systems, caregiver needs fit within the palliative care philosophy of treating the patient as part of a larger social system. Life satisfaction may be the most challenging to address, likely requiring interventions that target key constructs such as emphasizing the positive aspects of treatments to encourage optimism, using psychotherapeutic interventions to help patients achieve a sense of completion, and assisting patients with acceptance of their death. Since acceptance is individualistic, consideration must be given to individual preferences for ambiguity about the future and details on illness [68]. Finally, gender and mortality were risk factors in our study and others [2,9,25,84], but they are not modifiable. Their relevance lies in ensuring that resources are available to more frequently screen females and patients closer to death.

## Competing interests

The authors declare that they have no competing interests.

## Authors’ contributions

KF conceived of the study, designed the study, conducted the statistical analysis and interpretation of the data, and drafted the manuscript. DG assisted with the conception and design of the study, reviewed the statistical analysis and interpretation of the data, and critically revised drafts of the manuscript. HS reviewed the design of the study and interpretation of the data, and critically revised drafts of the manuscript. KB and SF critically revised drafts of the manuscript. All of those who critically revised drafts of the manuscript made important contributions to its intellectual content. TS was responsible for acquisition of the data, and reviewed and approved the final draft of the manuscript. All authors have read and given their final approval of the submitted manuscript.

## Pre-publication history

The pre-publication history for this paper can be accessed here:

http://www.biomedcentral.com/1472-684X/13/10/prepub

## Supplementary Material

Additional file 1: Table S1 Definition of independent variables and response categories [38,39,84]. Click here for file
