# Supplementary material for: Prevalence and risk factors of depressive symptoms in a Canadian palliative home care population: a cross-sectional study
Source: BMC Palliat Care. 2014 Mar 17;13:10. doi: 10.1186/1472-684X-13-10 (PMC4003817; doi:10.1186/1472-684X-13-10)
Supplement: Additional file 1: Table S1 — Definition of independent variables and response categories [38,39,84]. [file 1472-684X-13-10-S1.doc]

**Additional file 1: Table S1: Definition of independent variables and response c**ategories

| **Variable** | **Self Reported (SR), Assessor Reported (AR)** | **Derivation** | **Response Categories** |
| --- | --- | --- | --- |
| Age | SR | Direct^a^ | 1=18-64, 2= 65-74, 3=75-84, 4= 85+ (years) |
| Gender | AR | Direct | 1=Male, 2=Female |
| Marital Status | SR | Direct, Grouped into 2 categories | 2=Married (Married, Partner), 1 = Other (Never Married, Separated, Divorced, Widowed) |
| Site | AR | Direct, site # replaced name to protect confidentiality | Integer 1-6 |
| Co-morbidities (#) | AR | Total number of non-blank fields (up to 7 conditions can be recorded for patient) | Integer 1-7 |
| Primary Diagnosis | AR | Disease that is main reason for palliation, Grouped into 4 main conditions | 1=Cancer, 2=Cardiovascular,3=COPD, 4=Other |
| Awareness of Prognosis | AR | Direct, applies for patients with estimated prognosis < 6 months | 1 (Yes)=Aware, 0 (No)= Not aware |
| Prognosis | AR | Direct, Grouped into 3 categories | 1=Imminent (days) to less than 6 weeks, 2=Greater than 6weeks and less than 6 months, 3= Greater than6 months |
| CHESS^b^ | AR | Composite measure based on presence of health symptoms, decline in decision making, decline in ADL’s | 0-1(mild)=, 2-5(moderate-severe) |
| Pain Scale | AR | Composite measured based on frequency and intensity of pain (85) | 0 (mild)=no pain, 1 (mild)=less than daily pain, 2 (moderate)=daily pain minor, 3 (severe)=daily pain horrible/excruciating |
| CPS^c^ | AR | Composite measure based on daily Decision-Making (6 severity levels) and Memory Recall (2 severity levels) (38) | 0-1=intact, 2-4=mild-moderate impairment, 5-6=severe impairment |
| ADL SHS^d^ | AR | Composite measure based on 7 severity levels for 8 daily activities (e.g. bathing, personal hygiene, eating) (39) | 0=Independent, 1=Supervision, 2 = Limited, 3=Extensive 1, 4=Extensive 2, 5=Dependent, 6=Total Dependence |
| Communication Disorders | AR | Conditional measure, “Yes” indicates one of 3 disorders (expression, comprehension, hearing) present with severity 2+ (range 0-4), “No” indicates less severe or no disorders | 1 (Yes)=Communication disorders, 2 (No) =No/minor communication disorders |
| Sleep Disorders | AR | Composite (summative) measure based on too little sleep (5 severity levels) and too much sleep (5 severity levels) | Integer ranging 0 -8 (higher number indicating more sleep problems) |
| Appetite | AR | Direct (Ate ≤ 1 meal on 2 of last 3 days) | 0=No (Good) 1=Yes (Poor) |
| Dyspnea | AR | Direct | 1 (minimal)=Not present, absent at rest and present for moderate activities, 2 (moderate) =Absent at rest, present for day-to-day activities, 3 (severe) =present at rest |
| Gastrointestinal Problems | AR | Composite (summative) score based on 7 problems (e.g., acid reflux, constipation, vomiting) each having 5 severity levels | Integer ranging 0-28, 0-2=Minimal, 3-28=Moderate-Severe |
| Life Satisfaction | AR | Conditional measure, “High” Life Satisfaction indicates 5 conditions present (sense of completion, sense of progress towards completion, acceptance, possesses strengths, positive outlook) and “Low” Life Satisfaction indicates 1 or more of the 5 conditions are absent | 0=Low Life Satisfaction, 1= High Life Satisfaction |
| Living Alone | SR | Direct, Grouped into 2 categories | 1= Alone, 0=Not alone |
| Supportive Family | AR | Direct | 1 (Yes) =Supportive family, 0 (No) =No supportive family |
| Caregiver Distress | AR | Conditional measure, “Yes” indicates caregiver exhibits one of 3 problems (unable to continue, expresses distress, overwhelmed), “No” for all other responses | 1 (Yes)=caregiver exhibits signs of distress, 0 (No) = caregiver does not exhibit signs of distress |

^a”^Direct” means direct from an item in the interRAI PC, without re-coding or modification

**^b^** CHESS=Changes in Health, End-Stage Disease and Symptoms and Signs.

^c^ CPS=Cognitive Performance Scale.

^d^ADL SHS=Activities of Daily Living Self-performance Hierarchy Scale.
